# TWIST1 drives cisplatin resistance and cell survival in an ovarian cancer model, via upregulation of *GAS6*, *L1CAM*, and Akt signalling

**DOI:** 10.1038/srep37652

**Published:** 2016-11-23

**Authors:** Cai M. Roberts, Michelle A. Tran, Mary C. Pitruzzello, Wei Wen, Joana Loeza, Thanh H. Dellinger, Gil Mor, Carlotta A. Glackin

**Affiliations:** 1 Department of Developmental and Stem Cell Biology, 1500 E. Duarte Road Duarte, CA 91010, USA; 2 Irell and Manella Graduate School of Biological Sciences, Yale University School of Medicine, 333 Cedar Street, New Haven, CT 06510, USA; 3 Division of Reproductive Sciences, Yale University School of Medicine, 333 Cedar Street, New Haven, CT 06510, USA; 4 Department of Surgery, Division of Gynaecologic Oncology, City of Hope Medical Center, 1500 E. Duarte Road, Duarte, CA 91010, USA; 5 California State University, Los Angeles, 5151 State University Drive, Los Angeles, CA 90032, USA

## Abstract

Epithelial ovarian cancer (EOC) is the most deadly gynaecologic malignancy due to late onset of symptoms and propensity towards drug resistance. Epithelial-mesenchymal transition (EMT) has been linked to the development of chemoresistance in other cancers, yet little is known regarding its role in EOC. In this study, we sought to determine the role of the transcription factor TWIST1, a master regulator of EMT, on cisplatin resistance in an EOC model. We created two Ovcar8-derived cell lines that differed only in their TWIST1 expression. TWIST1 expression led to increased tumour engraftment in mice, as well as cisplatin resistance *in vitro*. RNA sequencing analysis revealed that TWIST1 expression resulted in upregulation of GAS6 and L1CAM and downregulation of HMGA2. Knockdown studies of these genes demonstrated that loss of GAS6 or L1CAM sensitized cells to cisplatin, but that loss of HMGA2 did not give rise to chemoresistance. TWIST1, in part via GAS6 and L1CAM, led to higher expression and activation of Akt upon cisplatin treatment, and inhibition of Akt activation sensitized cells to cisplatin. These results suggest TWIST1- and EMT-driven increase in Akt activation, and thus tumour cell proliferation, as a potential mechanism of drug resistance in EOC.

Epithelial ovarian cancer (EOC), which accounts for over 90% of ovarian tumours, is the most lethal gynaecologic malignancy^1^,^2^,^3^. A significant challenge in the treatment of EOC is the frequent development of tumour recurrence and chemoresistance. We and others have previously shown that ovarian cancer stem cells (CSCs) that survive initial rounds of chemotherapy facilitate this tumour recurrence^4^,^5^. A key factor in this reactivation of cancer stem cells is TWIST1, a transcription factor that is required for normal early mesoderm development but silenced in most adult tissues^6^,^7^,^8^. TWIST1 is reactivated in many cancers, where it drives an epithelial to mesenchymal transition (EMT), leading to metastasis^8^,^9^. In a variety of tumour types, TWIST1 has also been linked to angiogenesis, resistance to apoptosis, and cancer cell stemness^10^,^11^,^12^. In ovarian cancer, TWIST1 protein is degraded in CSCs, maintaining CSCs in an epithelial state. However, once TWIST1 protein expression persists, CSCs undergo EMT, leading to proliferation and metastasis^5^,^13^.

Despite its known role in activation of the stem cell pool, the direct role of TWIST1 in drug resistance in ovarian cancer is relatively unknown. Multiple downstream TWIST1 target genes have been implicated in drug resistance, including interleukin 8 and matrix metalloproteinases 2 and 9^14^,^15^,^16^. Additionally, TWIST1 has been shown to regulate Gli1, which upregulates the DNA repair protein ERCC1. ERCC1 is partially responsible for the repair of cisplatin-induced DNA crosslinks^17^,^18^. In other tumour types, TWIST1 has been linked to resistance to cisplatin, as well as paclitaxel and doxorubicin^19^,^20^. While the EMT process as a whole has previously been correlated with drug resistance in EOC, TWIST1 itself has never been causally linked^21^. The related transcription factor TWIST2 has been shown to lead to platinum resistance via Akt activation in another EOC model, but whether TWIST1 can function in the same manner is unknown^22^. Therefore, we sought to determine the distinct role of TWIST1 in cisplatin resistance in an EOC model. This study is the first to focus on the specific mechanisms by which TWIST1 confers cisplatin drug resistance in ovarian cancer, a novel function for a transcription factor that has previously primarily been only associated with tumour cell motility. Connecting TWIST1 and the EMT process as a whole to the dual malignant functions of increased cancerous cell proliferation and drug resistance makes it an especially attractive target for aggressive, drug-resistant carcinomas that require a combination of therapeutic approaches.

## Results

### Creation of Ovcar8 derived stable lines with differential *TWIST1* expression

**Figure 1:**
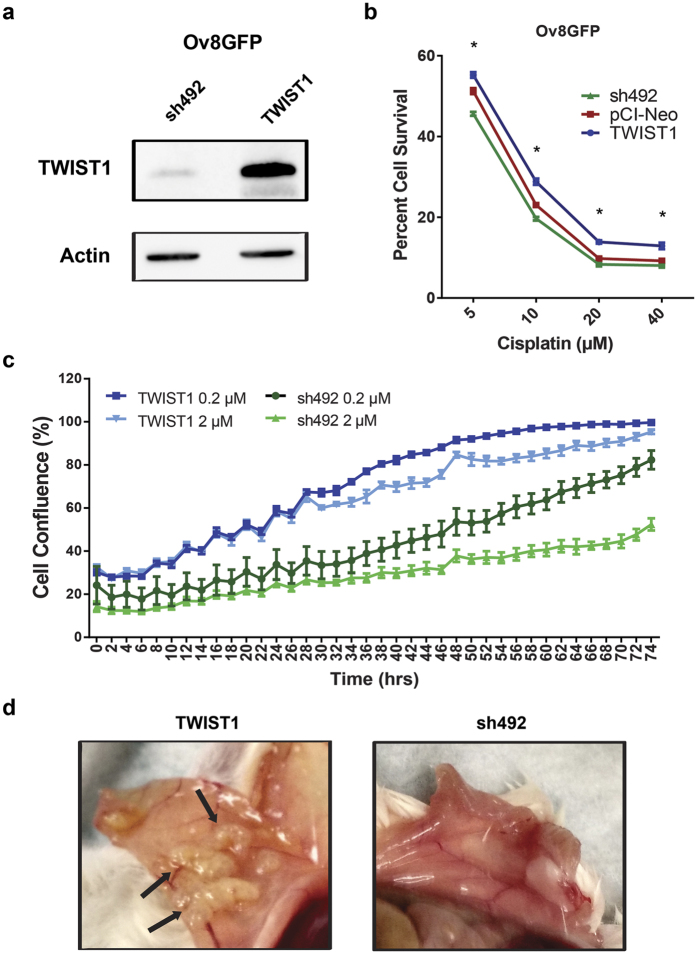
*TWIST1* overexpression leads to cisplatin resistance and enhanced tumour cell engraftment. (**a**) Western blotting demonstrates differential expression of *TWIST1* between Ov8GFP stably transfected cell lines. Blots cropped for clarity; full blots are shown in Supplementary Fig. S5. (**b**) SRB assay demonstrates that *TWIST1* expression leads to increased survival following exposure to cisplatin, particularly at lower doses (5, 10, and 20 μM, p < 0.0001; 40 μM, p = 0.0002). (**c**) Time lapse microscopy shows that across two logs of cisplatin doses, *TWIST1* expression leads to faster growth of cells. *TWIST1*-expressing cells achieve greater confluence over time than *TWIST1* knockdown cells at corresponding drug dose. Average slopes of the lines indicate a faster rate of growth for TWIST1 cells than sh492 cells until confluence is reached. Compare dark blue (slope = 1.15 over 48 hr) vs dark green (0.66 over 48 hr) and light blue (slope = 0.94 over 74 hours) vs light green (0.79 over 74 hr). (**d**) *In vivo* tumorigenesis assay shows that *TWIST1*-expressing cells give rise to widespread disseminated tumours, especially lining the wall of the peritoneal cavity. In contrast, sh492-expressing cells do not colonize the peritoneal wall. Arrows indicate carcinomatosis in mice engrafted with Ov8GFP-TWIST1 cells. All error bars represent standard error of the mean.

The ovarian cancer cell line Ovcar8 was transfected with a viral construct encoding an enhanced GFP-firefly luciferase fusion protein (CMV-p:EGFP-ffluc pHIV7) to make the Ov8GFP cell line, as we have described for previous cell line models^23^. We then transfected Ov8GFP cells with either TWIST1 or sh492, a previously validated shRNA against TWIST1^24^,^25^, using the pCI-Neo G418-selectable plasmid vector system. Following G418 selection of cells with stably integrated plasmid, we verified that TWIST1 was differentially expressed in the two cell lines – referred to hereafter as Ov8GFP-TWIST1 and Ov8GFP-sh492 – via western blot (Fig. 1a). Parental Ov8GFP cells express an intermediate level of TWIST1, thus an empty pCI-Neo vector resulted in intermediate TWIST1 expression, showing no substantial effect on TWIST1 from transfection alone (Fig. S1a,b). Reflecting their native *TWIST1* expression, Ovcar8-derived lines exhibited mesenchymal morphology (Fig. S2).

### *TWIST1* expressing cells are cisplatin resistant

We evaluated the effect of *TWIST1* expression in response to cisplatin. Following 72 hr incubation with cisplatin, sulphorhodamine B (SRB) cell survival assays showed that TWIST1-overexpressing cells exhibited greater survival than TWIST1 knockdown cells, normalized to untreated cells of each line (Fig. 1b). Cells transfected with empty pCI-Neo vector had intermediate survival compared to TWIST1 and sh492, confirming dose dependence of TWIST1 on cisplatin resistance (Fig. 1b). TWIST1 also affected the kinetics of cell growth during cisplatin treatment. Monitoring of cell confluence at 2 hr intervals showed that Ov8GFP-TWIST1 cells proliferated more rapidly than their sh492 counterparts (compare slope of light blue vs light green and dark blue vs dark green plots) when treated with 0.2 or 2 μM cisplatin (Fig. 1c and S2).

### *TWIST1*-expressing cells show enhanced engraftment in mice

We next evaluated the pro-survival and proliferation phenotype of TWIST1-overexpressing cells in tumour engraftment. We injected either Ov8GFP-TWIST1 or Ov8GFP-sh492 cells intraperitoneally into NSG mice (n=4 per group). Seven weeks after the injection, tumour burden and distribution in these mice were evaluated via pathological examination of haematoxylin and eosin stained tissue sections and images of the peritoneal cavity obtained at necropsy. All four mice that received Ov8GFP-TWIST1 developed ovarian tumours were graded 4/4 by a certified veterinary pathologist. By contrast, only two mice given Ov8GFP-sh492 exhibited ovarian tumours, only one of which was graded a 4 (Table 1). Three mice that received Ov8GFP-TWIST1 had detectable tumour masses within intra-abdominal organs (i.e. parenchymal tumour metastases), compared to only one of four mice in the Ov8GFP-sh492 group (Table 1). Additionally, necropsy images of mice show that mice engrafted with Ov8GFP-TWIST1 had many disseminated tumour masses in the intra-abdominal peritoneal lining, which were absent in the peritoneum of the mice engrafted with Ov8GFP-sh492 (Fig. 1d). This disseminated tumour distribution parallels the peritoneal surface carcinomatosis found frequently in advanced stage ovarian cancer patients. Taken collectively, these data strongly suggest a critical role for TWIST1 in promoting survival, proliferation, and engraftment of tumour cells in the ovaries and peritoneal space.

### RNA sequencing demonstrates differential expression of GAS6, L1CAM, and HMGA2

In order to determine which downstream pathways may be responsible for TWIST1-mediated proliferation, drug resistance, and cell survival, we performed RNA sequencing analysis. In addition to TWIST1 itself, a total of 51 genes were found to be differentially expressed between Ov8GFP-TWIST1 and –sh492 (>1.5 fold difference, p < 0.05), 18 downregulated by TWIST1 and 33 upregulated. As expected given TWIST1’s role in EMT during development and metastasis^8^, gene ontology (GO) terms enriched amongst TWIST1 regulated genes included Cell Movement and Cell Morphology. Additional enriched GO terms included Cellular Growth/ Proliferation and Cell Death and Survival. Ingenuity Pathway Analysis showed that apoptotic and migration signalling pathways intersect at TWIST1 and its target genes, including genes identified in our RNA sequencing results (Fig. S3). This finding suggests that TWIST1 may act to promote both proliferation and migration of tumour cells. A full list of differentially expressed genes is given in Table S1.

**Figure 2:**
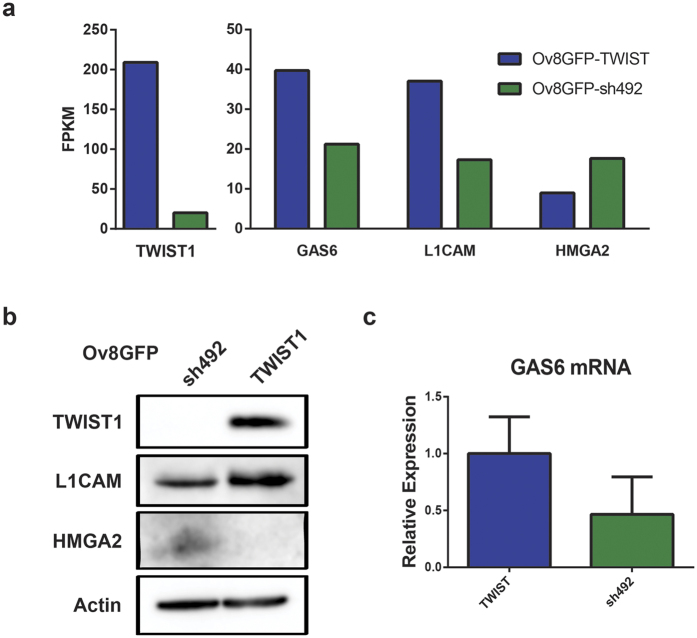
RNA sequencing reveals differential expression of *GAS6*, *L1CAM*, and *HMGA2*. (**a**) RNA sequencing showed approximately 2-fold increases in *GAS6* and *L1CAM* and a 2-fold decrease in *HMGA2* mRNA when *TWIST1* is overexpressed. FPKM, fragments per kilobase per million reads. (**b**) Western blot confirms differential expression of *L1CAM* and *HMGA2* found by RNA-seq. Blots cropped for clarity; full blots are shown in Supplementary Fig. S5. (**c**) No western blot was possible for GAS6, as the protein is secreted, but qRT-PCR shows on average a 50% decrease in *GAS6* mRNA level upon *TWIST1* knockdown. p = 0.31, although a clear trend is present. Error bars represent standard error of the mean.

As we were focused on the role of TWIST1 in drug resistance, we did not study any gene whose known function related only to development or cell migration. On the contrary, on the basis of their function in regulating cell survival, cell proliferation, and DNA repair, we selected *GAS6*, *L1CAM*, and *HMGA2* for further analysis. *GAS6* and *L1CAM* were upregulated approximately two fold in Ov8GFP-TWIST1 cells, while *HMGA2* was upregulated two fold in Ov8GFP-sh492 (Fig. 2a). We further verified that these genes were differentially expressed in our two cell lines. Western blot analysis confirmed that *L1CAM* was elevated and *HMGA2* reduced in Ov8GFP-TWIST1 cells, as compared to Ov8GFP-sh492 (Fig. 2b). Because GAS6 is secreted from cells, its expression was confirmed by qRT-PCR rather than western. Despite variability in expression in both Ov8GFP-TWIST1 and -sh492 cells, *TWIST1* expressing cells had two-fold higher levels of *GAS6* mRNA on average (Fig. 2c). We also found that tumours from mice given Ov8GFP-TWIST1 cells showed uniform IHC staining for *L1CAM*. Tumours from sh492 mice were heterogeneous, with areas in which staining was entirely absent (Fig. S3).

**Figure 3:**
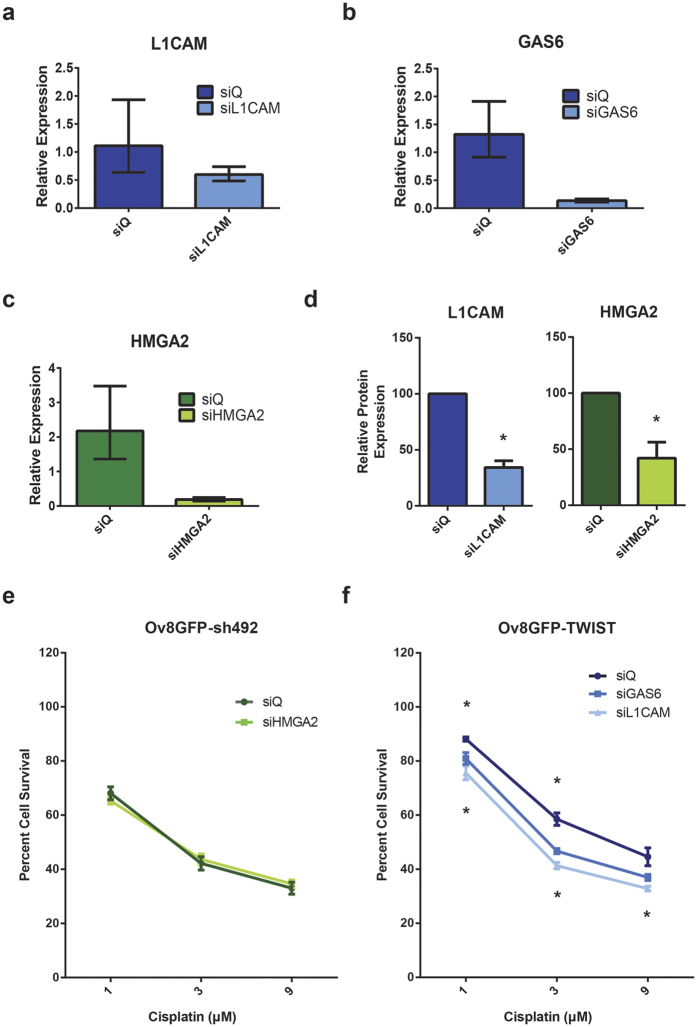
Knockdown of *GAS6* or *L1CAM* reverses drug resistance. (**a–c**) Validation of siRNAs targeting genes of interest. qRT-PCR confirms knockdown of *L1CAM* (46%) and *GAS6* (90%) in Ov8GFP-TWIST1 cells treated with corresponding siRNAs, and 91% knockdown of *HMGA2* in Ov8GFP-sh492 cells treated with *HMGA2* siRNAs. (**d**) Western blot confirms knockdown of *L1CAM* and *HMGA2* at the protein level (normalized results from three independent experiments, p = 0.0276 for *HMGA2*, p = 0.0042 for *L1CAM*). (**e**) SRB assay demonstrates that knockdown of *HMGA2* in Ov8GFP-sh492 cells is not sufficient to confer an increased resistance to cisplatin. (**f**) Knockdown of either *GAS6* or *L1CAM* in Ov8GFP-TWIST1 cells sensitizes this line to cisplatin, compared to treatment with non-targeting siRNA. Upper asterisks, siGAS6 (1 μM, p = 0.0108, 3 μM p = 0.00077, 9 μM p = 0.054); lower, siL1CAM (1 μM, p = 0.00077, 3 μM p < 0.0001, 9 μM p = 0.0064). qPCR error bars represent minimum and maximum values calculated by the StepOne software analysis. SRB error bars represent standard error of the mean.

We next knocked down each of these three genes to observe their individual effects on TWIST1-driven cell survival. qRT-PCR showed that siRNA against *L1CAM* and *GAS6* produced 46% and 90% knockdown of their target mRNAs, respectively, in Ov8GFP-TWIST1 cells compared to non-targeting control siRNA (siQ) (Fig. 3a,b). An siRNA pool against *HMGA2* reduced *HMGA2* mRNA levels by 91% on average in Ov8GFP-sh492 cells (Fig. 3c). Knockdown of *L1CAM* and *HMGA2* by their respective siRNA sequences was also confirmed at the protein level via western blot (Fig. 3d).

### *HMGA2* knockdown does not confer cisplatin resistance

As *HMGA2* is a negative regulator of ERCC1^26^, we hypothesized that knockdown of *HMGA2* might upregulate the DNA repair pathway responsible for the repair of the DNA crosslinks caused by cisplatin. Thus, we expected that *HMGA2* knockdown cells would show enhanced cisplatin resistance. However, an SRB cell survival assay showed that *HMGA2* knockdown had no impact on the proportion of Ov8GFP-sh492 cells able to survive cisplatin treatment (Fig. 3e). This may be due to the redundancy of DNA repair pathways or the compensatory activation of ERCC1 by additional factors; however, further studies are needed to determine if this is truly the case.

### Knockdown of *GAS6* or *L1CAM* sensitizes cells to cisplatin

We also hypothesized that knockdown of *GAS6* or *L1CAM* might sensitize cells to cisplatin due to abrogated survival signalling downstream from these factors. In order to test this hypothesis, we performed an SRB assay on Ov8GFP-TWIST1 cells treated with siQ or siRNA pools against *GAS6* or *L1CAM*. We found that knockdown of either gene was able to sensitize cells to cisplatin, with *L1CAM* knockdown reducing cell survival by up to 20% (Fig. 3f).

### *TWIST1, GAS6*, and *L1CAM* upregulate expression and phosphorylation of Akt in response to cisplatin

Given that both *GAS6* and *L1CAM* have been linked to Akt signalling^27^,^28^, and that TWIST2-mediated activation of Akt has been previously implicated in acquired cisplatin resistance^22^, we hypothesized that Akt may also be a key factor downstream from *TWIST1, GAS6,* and *L1CAM*. We also hypothesized that knockdown of *GAS6* or *L1CAM* in *TWIST1* overexpressing cells could inhibit upregulation and activation of Akt.

**Figure 4:**
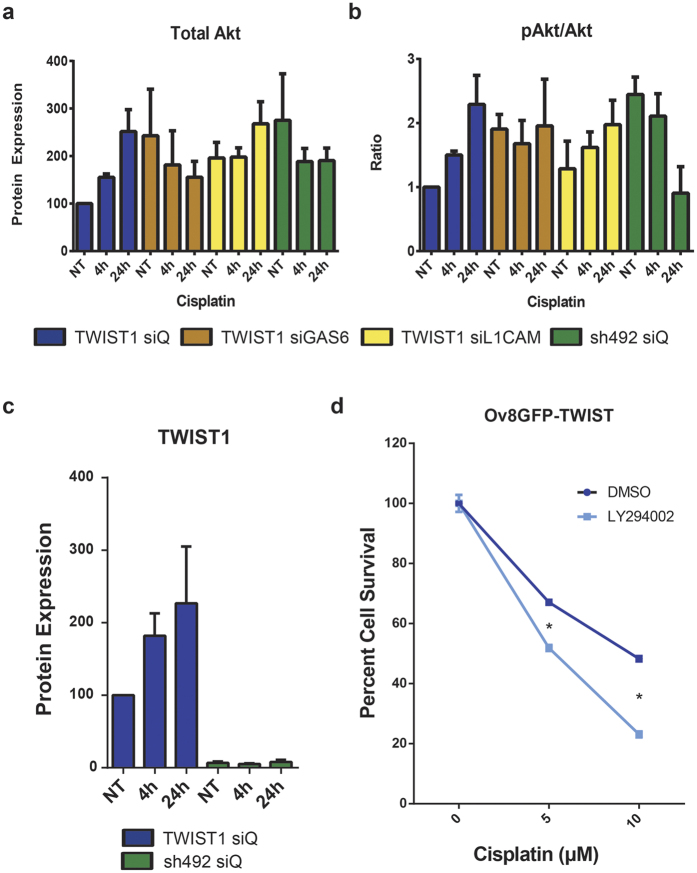
*TWIST1*, *GAS6*, and *L1CAM* expression lead to upregulation of Akt signalling following cisplatin treatment. (**a**) Quantification of western blot data shows that over the course of 24 hr, 5 μM cisplatin treatment leads to increased levels of Akt in Ov8GFP-TWIST1 cells (blue), but not in Ov8GFP-sh492 cells (green). Knockdown of *GAS6* (brown) or *L1CAM* (yellow) in Ov8GFP-TWIST1 cells partially abrogates the increase in Akt. NT, not treated with cisplatin. (**b**) Western blot also reveals an increase in activation of Akt via phosphorylation at Ser 473 over 24 hr of 5 μM cisplatin in *TWIST1* expressing cells (blue). The opposite is true in Ov8GFP-sh492 cells, in which Akt activity is reduced over the same time period (green). Knockdown of GAS6 in Ov8GFP-TWIST1 cells (brown) maintains a constant pAkt/Akt ratio, while *L1CAM* knockdown (yellow) partially prevents Akt activation. (**c**) Ov8GFP-TWIST1 cells further increase their *TWIST1* expression up to 2.3 fold over 24 hr of exposure to 5 μM cisplatin (p=0.0827), whereas Ov8GFP-sh492 cells show no increase. (**d**) Treatment of Ov8GFP-TWIST1 cells with the PI3K inhibitor LY294002 to prevent Akt activation sensitized cells to cisplatin, compared to DMSO only control, supporting the assertion that Akt signalling is central to TWIST1-driven cisplatin resistance. p < 0.0001 for both concentrations. Error bars represent standard error of the mean.

Following treatment of cells with siQ control siRNA or pooled siRNAs against *GAS6* or *L1CAM*, western blotting of total Akt showed that while Ov8GFP-TWIST1 cells have lower initial Akt expression compared to OvGFP-sh492, continued exposure to 5 μM cisplatin led to a 150% increase in Akt levels in Ov8GFP-TWIST1 cells over the course of 24 hr (Fig. 4a). In Ov8GFP-sh492 cells, total Akt levels remain relatively constant over 24 hr of cisplatin exposure (Fig. 4a). Interestingly, the proportion of Akt in its active form (i.e. phosphorylated at Ser 473) increases 128% over the course of 24 hr in Ov8GFP-TWIST1 cells, even when normalized to total Akt expression at each time point (Fig. 4b). Conversely, OvGFP-sh492 cells show a 63% reduction in phosphorylated Akt over the same 24 hr period (Fig. 4b). The pattern of Akt activation in these cell lines mirrors the activation of *TWIST1* itself in Ov8GFP-TWIST1 cells over 24 hours of cisplatin treatment, which is absent in sh492 cells (Fig. 4c).

Western blotting also revealed that knockdown of either *GAS6* or *L1CAM* could partially prevent Akt upregulation, as it resulted in largely constant Akt levels over time (Fig. 4a). Similarly, knockdown of either *GAS6* or *L1CAM* produced levels of Akt phosphorylation intermediate between those seen in Ov8GFP-TWIST1 and OvGFP-sh492 cells treated with siQ control. *GAS6* knockdown kept the proportion of active Akt relatively constant, while loss of *L1CAM* led to increasing Akt activation at each time point, but only 53% over 24 hr, as compared to 128% for siQ treated Ov8GFP-TWIST1 cells (Fig. 4b).

### Inhibition of Akt activation sensitizes cells to cisplatin

In order to confirm that Akt mediates cisplatin resistance downstream of TWIST1 in our system, we treated Ov8GFP-TWIST1 cells with either cisplatin alone, or cisplatin plus the PI3K inhibitor LY294002, which prevents the phosphorylation of Akt by PI3K. Cells treated with the combination exhibited 15% greater cell death at 5 μM cisplatin and 25% greater cell death at 10 μM, compared to those treated with cisplatin alone, confirming that loss of Akt activation leads to cisplatin sensitivity in our model (Fig. 4d). This link between TWIST1 and Akt function, combined with the tumour engraftment data presented in Fig. 1, suggests that TWIST1-mediated cisplatin resistance may be a part of an overall increase in cell growth and proliferation signalling.

## Discussion

Epithelial ovarian cancer is characterized by tumours that are widely disseminated throughout the peritoneal cavities of patients and that have a high tendency for recurrence. Both of these unwanted phenotypes are made possible by the presence of cancer stem cells. As these cells are quiescent, drugs such as paclitaxel and cisplatin that target rapidly proliferating cells have little efficacy. Unfortunately once the bulk of the tumour mass has been eliminated by surgery and chemotherapy, CSCs drive cancer recurrence by re-entering the cell cycle and differentiating. We have previously shown that during this process of re-entry, the CSCs lose expression of CD44 and MyD88 and acquire mesenchymal characteristics^5^,^13^ due to the persistence of TWIST1 protein.

A growing body of studies link TWIST1 to many cancer processes outside of its traditionally studied roles in cell migration and metastasis. This current study examines additional cancer phenotypic impacts of TWIST1 in the context of differentiated EOC cells. We sought to determine the role TWIST1 plays in the acquisition of drug resistance in recurrent tumour cells. Prior studies have linked TWIST1 and the related protein TWIST2 to drug resistance in multiple tumour types, including ovarian, but the specific mechanism by which TWIST1 drives resistance in EOC is not well understood. To elucidate this mechanism, we created a pair of cell lines in the Ovcar8 background which differed in expression of *TWIST1*. We then employed SRB cell survival assays and IncuCyte cell growth studies to monitor the effects of TWIST1 and its target genes, providing both a static and dynamic measurement of cell proliferation, and using assays well suited to the Ovcar8 line.

In our EOC model, we found that sustained *TWIST1* overexpression in EOC cells with a mesenchymal phenotype led to enhanced cell survival and proliferation, both in the *in vivo* tumour engraftment assays and in the presence of cisplatin *in vitro* (Fig. 1). RNA-sequencing analysis of *TWIST1*-overexpressing and *TWIST1*- knockdown cells revealed 51 significantly differentially expressed genes. As expected given TWIST1’s well documented role in modulating cell migration and cell-extracellular matrix interactions, several of the genes identified related to these processes (Table S1). However, a number of genes also related to cell survival and proliferation signalling. Of these, we selected *GAS6, L1CAM*, and *HMGA2* for further study. We verified differential expression of these genes in our Ov8GFP-TWIST1 and -sh492 cell lines (Fig. 2), and validated a pool of siRNAs against each (Fig. 3). *HMGA2* has previously been linked to *TWIST1*, but in metastatic breast cancer, in which *TWIST1* and *HMGA2* are both targets of microRNA miR-33b^29^. *HMGA2* is a negative regulator of the nucleotide excision repair (NER) protein ERCC1, which is involved in the repair of platinum-induced DNA crosslinks^18^,^26^. We therefore hypothesized that knockdown of *HMGA2* would allow for cisplatin-induced upregulation of NER as previously reported^18^, and that *HMGA2* knockdown in Ov8GFP-sh492 cells would give rise to cisplatin resistance. However, we found that loss of *HMGA2* did not have any effect on cell survival in response to cisplatin (Fig. 3e). This may be due to redundancy of DNA repair signalling, as multiple factors will likely regulate NER, including Gli1^18^, and knockdown of a single regulatory protein may not be sufficient to impact NER function.

We next determined the impact of *GAS6* and *L1CAM* in the response to cisplatin. *GAS6* is known to have anti-apoptotic functions, and its receptor, Axl, is a known target of TWIST1, and a part of the EMT process^30^,^31^,^32^,^33^. Furthermore, *GAS6* has been linked to activation of Akt in muscle cells, and another study determined that Akt was required for the anti-apoptotic function of *GAS6*^27^,^31^. In addition, *GAS6* is a marker for poor prognosis in ovarian cancer, and Axl is thought to be essential for metastatic EOC^34^,^35^. Together, these studies suggest that *GAS6* may contribute to the cell survival phenotype observed in our EOC cells.

*L1CAM* has also been linked to the EMT process and metastasis^36^,^37^. The TWIST1 target gene *Slug* has been shown to regulate *L1CAM* expression in cancer^38^,^39^. Moreover, *L1CAM* has been linked to resistance to apoptosis in both intestinal tissue and an EOC cell line, and to drug resistance in pancreatic cancer^40^,^41^,^42^. Like *GAS6, L1CAM* has also attracted interest as a biomarker in gynaecologic cancers. It was found to be an indicator of poor survival in endometrial carcinoma and correlated with progression, stage, grade, and poor survival in ovarian cancer^43^,^44^. Finally, like *GAS6, L1CAM* has been shown to signal through the PI3K/Akt pathway, and drives progression and invasion in gallbladder carcinoma^28^. We therefore examined whether knockdown of *GAS6* or *L1CAM* would impact cisplatin treatment efficacy. We found that knockdown of either gene was able to sensitize TWIST1 overexpressing cells to cisplatin (Fig. 3f).

As outlined above, both *GAS6* and *L1CAM* have been linked to Akt signalling, and the Twist family member *TWIST2* was recently found to drive cisplatin resistance via Akt^22^. Moreover, TWIST1-mediated activation of Akt2 led to drug resistance in breast cancer^19^. Loss of Akt reversed platinum resistance in the A2780 ovarian cancer model^45^, although A2780 has been shown to lack many of the genetic hallmarks of high grade serous EOC^46^. We therefore sought to determine if *TWIST1*, along with *GAS6* and *L1CAM*, was acting via Akt. We found that expression of *TWIST1* led to an increase in Akt expression and activation over the course of 24 hr of cisplatin treatment *in vitro*, while *TWIST1* knockdown led to a decrease in Akt activity (Fig. 4a,b). Knockdown of *GAS6* or *L1CAM* partially prevented Akt activation in Ov8GFP-TWIST1 cells (Fig. 4a,b). As expected, inhibition of Akt activation by the PI3K inhibitor LY294002 substantially sensitized OV8GFP-TWIST1 cells to cisplatin, even after correcting for the anti-proliferative effects of the inhibitor (Fig. 4c).

**Figure 5:**
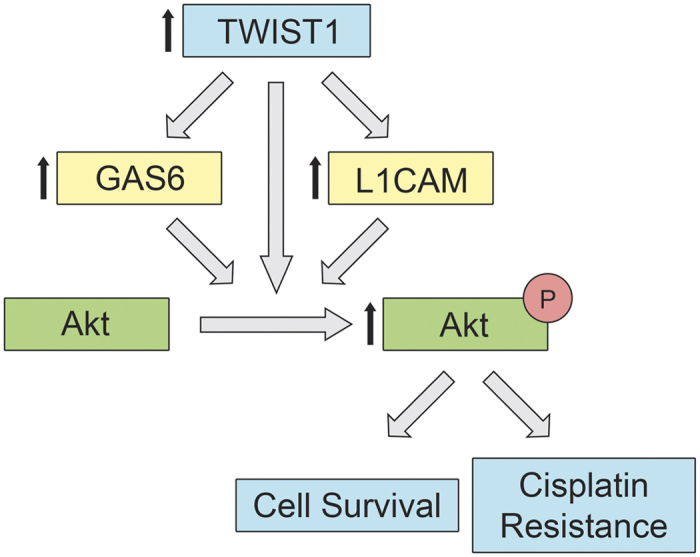
Schematic representation of our proposed model. *TWIST1* upregulates expression of *GAS6* and *L1CAM. TWIST1*, *GAS6*, and *L1CAM* facilitate phosphorylation and activation of Akt, leading to increased proliferation. This in turn results in cisplatin resistance and greater tumour cell engraftment *in vivo*.

Taken together, our data suggest a model where TWIST1-mediated upregulation of *L1CAM* expression and *GAS6*/Axl signalling lead to higher throughput of Akt signalling. This increase in proliferation gives rise to greater cell survival during tumour cell engraftment assays. This model also suggests that TWIST1-mediated drug resistance is a result of increased proliferation, rather than direct inhibition of cisplatin activity by DNA repair proteins or upregulation of drug efflux, for example by downregulation of HMGA2 in TWIST1 overexpressing cells (Fig. 5). This interpretation is also supported by the known role of TWIST1 in the activation of the quiescent CSC pool to give rise to disseminated, recurrent tumours^13^.

This is the first study on the role TWIST1 plays in acquired drug resistance in ovarian cancer. The current study benefits from the design of our cell lines, which allow us to focus only on the effects of TWIST1 and its target genes, without the confounding variables of additional genetic changes between drug-sensitive and resistant cells. Nevertheless, our study employs only one such pair and our findings must still be generalized to additional cell lines and tumour types.

Additional work will also be needed to refine our mechanistic model. Moreover, there is little data available on the intermediate factors connecting *L1CAM* to Akt, though *GAS6*-mediated survival signalling was shown to depend on NF-κB^30^, which we and others have shown is a TWIST1 partner protein^25^. In addition, knockdown of *L1CAM* and *GAS6* only partially prevented Akt activation, suggesting that additional factors downstream of TWIST may also lead to increased proliferation in TWIST1-expressing cells. There are many additional genes that were differentially expressed between Ov8GFP-TWIST1 and –sh492 cells (Table S1), and these genes may also be involved in the regulation of Akt signalling or drug response in these cell lines.

Further study of the roles played by TWIST1 in EOC will be vital in our efforts to overcome the challenges of drug resistance and metastasis during recurrence. Even TWIST1’s role in cisplatin resistance is complex; TWIST1 is constitutively degraded in the epithelial cancer stem cells, widely considered to be the most drug resistant subpopulation^13^. Nevertheless, we have shown evidence here that amongst the more mesenchymal differentiated cells, TWIST1 may actually lead to greater resistance to cisplatin. Future work by our laboratories will focus on TWIST1 function throughout the process of recurrence, including the mechanism by which TWIST1 is degraded in CSCs. Determining the cellular machinery responsible for TWIST1 protein turnover in EOC may shed light not only on its regulation in other cancers undergoing EMT, but also on its regulation during mesodermal development. Given its well-characterized function in promoting metastasis, we will also investigate the role of TWIST1 in the spread of EOC cells throughout the peritoneal space, from primary tumours of the ovary, the fallopian tube epithelium, and pockets of CSCs during recurrence.

Of great interest in multiple cancer types will be understanding and targeting the molecular pathways connecting proliferative and pro-metastatic signals in tumours that have undergone EMT. TWIST1 and EMT as a whole have been linked to a growing list of cancer phenomena, including angiogenesis, stem cell maintenance, and drug resistance. A recent meta-analysis showed that expression of EMT transcription factors was a significant risk factor for metastatic breast cancer patients, particularly those of Asian descent^47^. Fortunately, therapies designed to inhibit EMT and TWIST1 have shown promise. Expression of the tumour suppressor tristetraprolin (TPP), an inhibitor of TWIST1 and Snail, reversed mesenchymal phenotype in ovarian, lung, and colon cancer^48^. TPP also reduced the growth of ovarian cancer cells in culture^48^. Furthermore, an inhibitor of STAT3 reduced downstream activity of TWIST1, which led to impeded migration and invasion of melanoma cells^49^. Our group has also demonstrated the efficacy of TWIST1 siRNA in reducing melanoma growth *in vivo* via inhibition of apoptosis^11^.

Despite these successes, the signals that determine which, if any, EMT-linked processes are active in a given tumour remain unknown. A deeper understanding of EMT signalling may allow for the identification of additional novel therapeutic targets and strategies, in addition to reduction of TWIST1 itself, for the most aggressive types and subtypes of ovarian and other cancers.

## Methods

### Cell lines

Ovcar8 cells were obtained from ATCC, and engineered to stably express a GFP-firefly luciferase fusion protein, using the CMV-p:EGFP-ffluc pHIV7 vector (a gift from Christine Brown at City of Hope, as has been described previously^23^) to make the Ov8GFP line. The *TWIST1* gene or an shRNA targeting *TWIST1*, sh492, were cloned into the pCI-Neo vector from Promega (Madison, WI). Empty pCI-Neo vector was used as control. Lipofectamine 2000 (Thermo Fisher, Waltham, MA) was used to transfect the vectors into Ov8GFP cells and cells were treated with 0.8 mg/mL G418 (Sigma Aldrich, St. Louis, MO) to select for stable plasmid integration. Resulting cell lines are herein referred to as Ov8GFP-TWIST1, Ov8GFP-pCI-Neo, and Ov8GFP-sh492.

### Cell culture

All cells were grown in RPMI 1640 medium (Genesee Scientific, San Diego, CA) supplemented with 10% foetal bovine serum and 1% penicillin/streptomycin, in a tissue culture incubator maintaining 37 °C, 5% CO_2_, 90% humidity. Ov8GFP-TWIST1, Ov8GFP-pCI-Neo, and Ov8GFP-sh492 were grown in 0.4 mg/mL G418 to maintain TWIST1/pCI-Neo/sh492 plasmid integration. Cells were passaged every 2–4 days. Confluent cells were washed with PBS, detached with 0.25% trypsin-EDTA (Genesee Scientific), and transferred to new dishes.

### Gene knockdown

Small interfering RNA (siRNA) was used for knockdown of *GAS6*, *L1CAM*, and *HMGA2*. Lipofectamine 2000 was used to transfect siRNA into cells in OptiMEM medium (Thermo Fisher). Medium was changed to normal medium after 4 hr or on the following day. Non-targeting control siRNA, siQ, was AllStars Negative Control siRNA from Qiagen (Valencia, CA). Pooled siRNAs against *GAS6*, *L1CAM*, and *HMGA2* were obtained from Santa Cruz Biotechnology (Dallas, TX; item numbers sc-35450, sc-43172, and sc-37994, respectively).

### qRT-PCR

Total RNA was isolated from pelleted cells using the RNeasy Plus kit from Qiagen according to the manufacturer’s protocol. cDNA was reverse transcribed using the iScript cDNA Synthesis kit from Bio-Rad (Hercules, CA). Real time PCR was run either on an Applied Biosystems StepOnePlus machine using SYBR Select Master Mix (Life Technologies, Carlsbad, CA) or on a Bio-Rad iQ5 system using SYBR master mix from Kapa Biosystems (Wilmington, MA) in 20 μL reactions, in triplicate. Melt curves were obtained for all reactions. β-Actin was used as the endogenous control. Expression was determined using the 2^−ΔΔCt^ method. Primers used were: GAS6 Fwd 5’-CTGCATCAACAAGTATGGGTCTCCGT-3’, GAS6 Rev 5’-GTTCTCCTGGCTGCATTCGTTGA-3’, HMGA2 Fwd 5’-CAGCGCCTCAGAAGAGAGGACG-3’, HMGA2 Rev 5’-CCGTTTTTCTCCAGTGGCTTCTGCT-3’, L1CAM Fwd 5’-GCAGCAAGGGCGGCAAATACTCA-3’, L1CAM Rev 5’-CTTGATGTCCCCGTTGAGCGAT-3’, β-Actin Fwd 5’-CCGCAAAGACCTGTACGCCAAC-3’, β-Actin Rev 5’-CCAGGGCAGTGATCTCCTTCTG-3’.

### Western blotting

Cells were pelleted and washed once with phosphate buffered saline (PBS), then lysed in RIPA buffer. Protein concentration was determined using a BCA assay (Thermo Fisher). Equal masses of protein were run on 4% stacking, 10% resolving polyacrylamide gels, and then transferred to PVDF membrane (GE Healthcare Bio-Sciences, Pittsburgh, PA) using the Trans-Blot SD Semi-Dry Transfer Cell (Bio-Rad). Membranes were blocked in dry milk dissolved in PBS with shaking for 1 hr at room temperature or overnight at 4 °C. 3% BSA was used for blocking prior to select pAkt blots to reduce background signal. Primary and secondary antibodies were diluted as indicated below in 5% dry milk in PBS with 0.1–0.2% Tween-20. Incubation was performed at room temperature for one hr or overnight at 4 °C for primary, and at room temperature for one hr for secondary. Each antibody incubation was followed by five 5 min washes in PBS with 0.1% Tween-20. FemtoGlow hrP substrate (Michigan Diagnostics, Royal Oak, MI) and the Pxi4 chemiluminescent imager (Syngene, Frederick, MD) were used to acquire digital images. ECL 2 (Thermo Fisher) and Blue Devil Film (Genesee Scientific) were used to acquire film images. FemtoGlow was diluted 6-fold in water for imaging of actin bands. Membranes were stripped using Restore Western Blot Stripping Buffer (Thermo Fisher), rinsed twice in PBS, and the process was repeated for each protein tested. Antibodies used were TWIST1 (TWIST 2c1a, Santa Cruz Biotechnology sc-81417, 1:500), β-Actin (Sigma Aldrich A1978, 1:5,000), Phospho Akt (Ser473) (Cell Signaling Technology 9271, 1:1000), Akt (Cell Signaling Technology 9272, 1:1000), HMGA2 (HMGI-C 2421C6a, Santa Cruz Biotechnology sc-130024, 1:500), and L1CAM (NCAM-L1 D5, Santa Cruz Biotechnology sc-374046, 1:1000). For select digital and film westerns, bands were quantified using GeneTools software from Syngene or Image Studio Lite from LiCor (Lincoln, NB), respectively, and normalized to β-Actin.

### RNA sequencing analysis

RNA from biological replicates of Ov8GFP-sh492 and –TWIST1 was obtained as described for qRT-PCR. Quality was verified by absorption spectra using a NanoDrop 1000 spectrophotometer (Thermo Fisher). RNA sequencing of two samples per cell line was performed in triplicate using the Illumina Hi-seq platform by the Integrative Genomics Core facility at City of Hope. Data were analysed using the online Galaxy platform. Pipeline consisted of the following algorithms: Tophat (for alignment of sequenced fragments to the human genome), Cufflinks (for the assembly of aligned fragments into transcripts), Cuffmerge (for the merging of several Cufflink assemblies into a single file), and Cuffdiff (for determining differences in expression using a two-sample t-test). Ingenuity Pathway Analysis was used to build relationships and potential links to canonical signalling pathways based on previously published publications.

### Sulphorhodamine B assays

Cells were plated at 5,000 per well in normal medium in 96 well plates (100 μL/well), n=6 per drug concentration, per condition. Cells were allowed to adhere overnight, and then cisplatin was added. Cisplatin was prepared at 2x concentration in 100 μL normal medium, and then added to cells to yield 200 μL at 1x. Following 3 day incubation with cisplatin, medium was removed and cells were fixed with 10% trichloroacetic acid (TCA, 100 μL/well) at 4 °C for 1 hr. TCA was then removed and wells were rinsed with 200 μl water and allowed to air dry 10 min. Next, cells were incubated in 0.4% sulphorhodamine B (SRB) in 1% acetic acid at room temperature for 15 minutes, after which dye was discarded and wells were rinsed 3–4 times with 1% acetic acid until wash showed no further colour. Plates were air dried and excess SRB adhered to the walls of the wells was removed with a cotton swab. Finally, SRB was solubilized in 10 mM Tris base (200 μL/well) and absorbance was measured at 570 nm on a SpectraMax Plus plate reader (Molecular Devices, Sunnyvale, CA). Readings for each condition were normalized to untreated wells from the same condition. Cisplatin concentrations were adjusted to fit experimental demands. Lipofectamine transfected cells were more sensitive to cisplatin, thus a reduced cisplatin concentration across all conditions was required. For Akt studies, the PI3K inhibitor LY294002 was used to inhibit Akt activation (Fig. 4), and was obtained from Cell Signaling Technology (Danvers, MA).

### Real-time monitoring of cell confluence

In order to determine the effects of TWIST1 expression and cisplatin on the kinetics of cell growth, the IncuCyte ZOOM system (Essen BioScience, Ann Arbor, MI) was used as described previously^4^,^50^,^51^. Briefly, Ov8GFP-TWIST1 and Ov8GFP-sh492 cells were plated at 4,000 cells/well of a 96 well plate in quadruplicate overnight in normal medium. The following day, they were treated with 0.2 or 2 μM cisplatin (Teva Pharmaceuticals USA, Sellersville, PA) and imaged every two hr for 74 hr to determine cell confluence over time.

### *In vivo* tumorigenesis study

To determine the effects of TWIST1 on tumour engraftment and proliferation *in vivo*, 3.2 million Ov8GFP-TWIST1 or Ov8GFP-sh492 cells were injected intraperitoneally into female NOD.Cg-*Prkdc*^*scid*^*Il2rg*^*tm1Wjl*^*/*SzJ (NSG) mice (The Jackson Laboratory, Bar Harbor, ME). Four mice received each cell line. Tumours were allowed to grow for seven weeks, and tumour burden was evaluated at necropsy. Images were taken of mice, and peritoneal organs were harvested and fixed in formalin for evaluation by a board-certified veterinary pathologist with over 30 years of experience in pathology of experimental mouse models. Evaluation was done without knowledge of treatment group or experimental design. The amount of tumour growth within a tissue was graded using a progressive, semi-quantitative, tiered scale of 0–4, where 0 = no tumour growth and 4 = major portion of the tissue occupied by tumour growth. This study was conducted in accordance with a protocol approved by the Institutional Animal Care and Use Committee at the City of Hope Beckman Research Institute (Protocol no. 15002, approved 22 March 2016). Care was taken to minimise the number of mice used and the pain and discomfort of mice in the study.

### Statistics

Statistical significance between conditions for cytotoxicity assays was determined by a series of unpaired Student t-tests comparing TWIST1 to sh492 or comparing gene knockdown conditions to siQ control. The Holm-Sidak method was used to correct for multiple comparisons. No assumption of equal standard deviation was made. Knockdown of *HMGA2* and *L1CAM* protein was analysed using paired, one-sided t-tests. All calculations were done using Prism 6 (GraphPad Software, La Jolla, CA). Asterisks denote statistical significance (p < 0.05). Exact p-values and error bar parameters are given in figure legends. OneStep qPCR data, RNA-seq output, and slopes of IncuCyte graphs are not open to statistical analysis by our software, and so trends are numerically described in the text for these experiments.

## Additional Information

**How to cite this article**: Roberts, C. M. *et al.* TWIST1 drives cisplatin resistance and cell survival in an ovarian cancer model, via upregulation of *GAS6, L1CAM*, and Akt signalling. *Sci. Rep.* **6**, 37652; doi: 10.1038/srep37652 (2016).

**Accession Code**: RNA sequencing data is available online in the NCBI Gene Expression Omnibus, accession code GSE84425.

**Publisher’s note:** Springer Nature remains neutral with regard to jurisdictional claims in published maps and institutional affiliations.

## Supplementary Material

Supplementary Information

## Figures and Tables

**Table 1:** Results of pathology analysis.

Mouse	Tumour Score				
	Liver	Uterus	Ovary	Kidney	Spleen
sh492 A	0	0	4	0	0
sh492 B	1	0	2	0	0
sh492 C	0	0	0	0	0
sh492 D	0	—	—	0	0
TWIST1 A	0	0	4	0	0
TWIST1 B	0	—	4	0	1
TWIST1 C	1	0	4	0	0
TWIST1 D	1	0	4	0	0

*TWIST1* overexpressing cells gave rise to large ovarian tumours in 4/4 mice, whereas sh492 expressing cells gave rise to tumours in 2/4 mice, with only one matching the severity seen in TWIST1 tumours (1/4 sh492 scored 4 vs 4/4 TWIST1 scored 4). 3/4 mice receiving TWIST1-expressing cells developed a metastatic lesion in their liver or spleen, compared to 1/4 sh492 mice. A, B, C, and D refer to individual mice. “0” reflects a tumour score of 0, while “—” denotes no sample collected.
