# Supplementary material for: TWIST1 drives cisplatin resistance and cell survival in an ovarian cancer model, via upregulation of GAS6, L1CAM, and Akt signalling
Source: Sci Rep. 2016 Nov 23;6:37652. doi: 10.1038/srep37652 (PMC5120297; doi:10.1038/srep37652)
Supplement: Supplementary Information [file srep37652-s1.pdf]

Supplementary Information

Title:

TWIST1 drives cisplatin resistance and cell survival in an ovarian cancer model, via upregulation of GAS6, L1CAM, and Akt signalling

Authors:

Cai M. Roberts, Michelle A. Tran, Mary C. Pitruzzello, Wei Wen, Joana Loeza, Thanh H. Dellinger, Gil Mor, and Carlotta A. Glackin

Figure S1

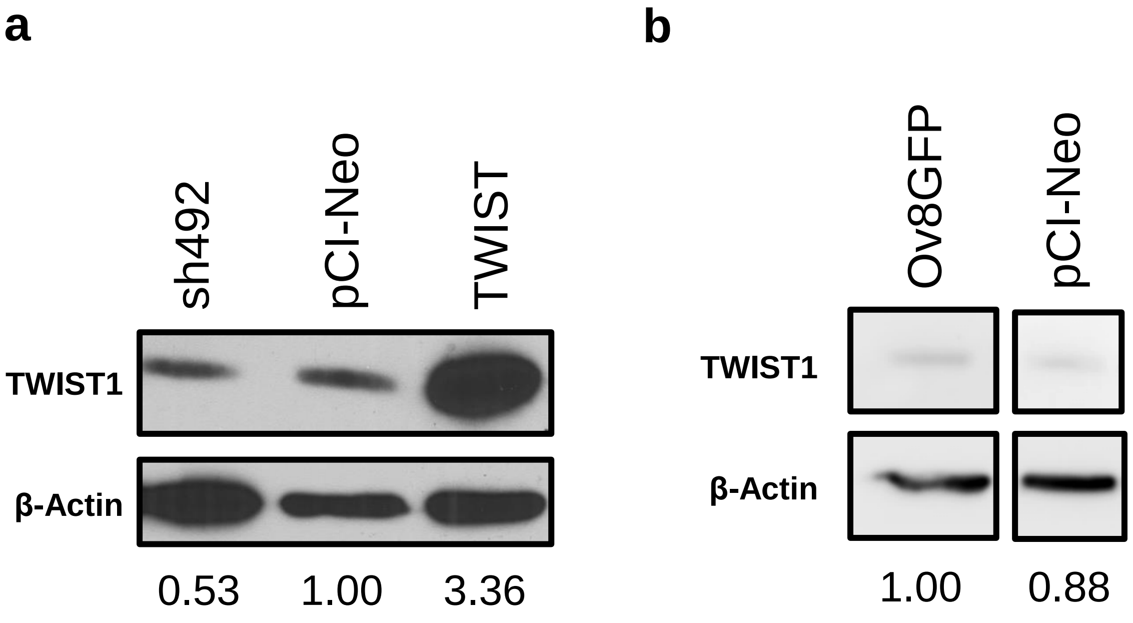

Figure S1. Cells transfected with empty pCI-Neo vector exhibit intermediate phenotype. **a.** Quantification of representative western blot data demonstrating that TWIST1 expression is reduced 2-fold by sh492 and increased approximately 3.5-fold by expression of a second copy of the gene. **b.** Transfection of cells with empty pCI-Neo vector does not substantially affect TWIST1 expression levels. Representative western data shown. Blots cropped for clarity; full blots are shown in Supplementary Figure S5.

Figure S2

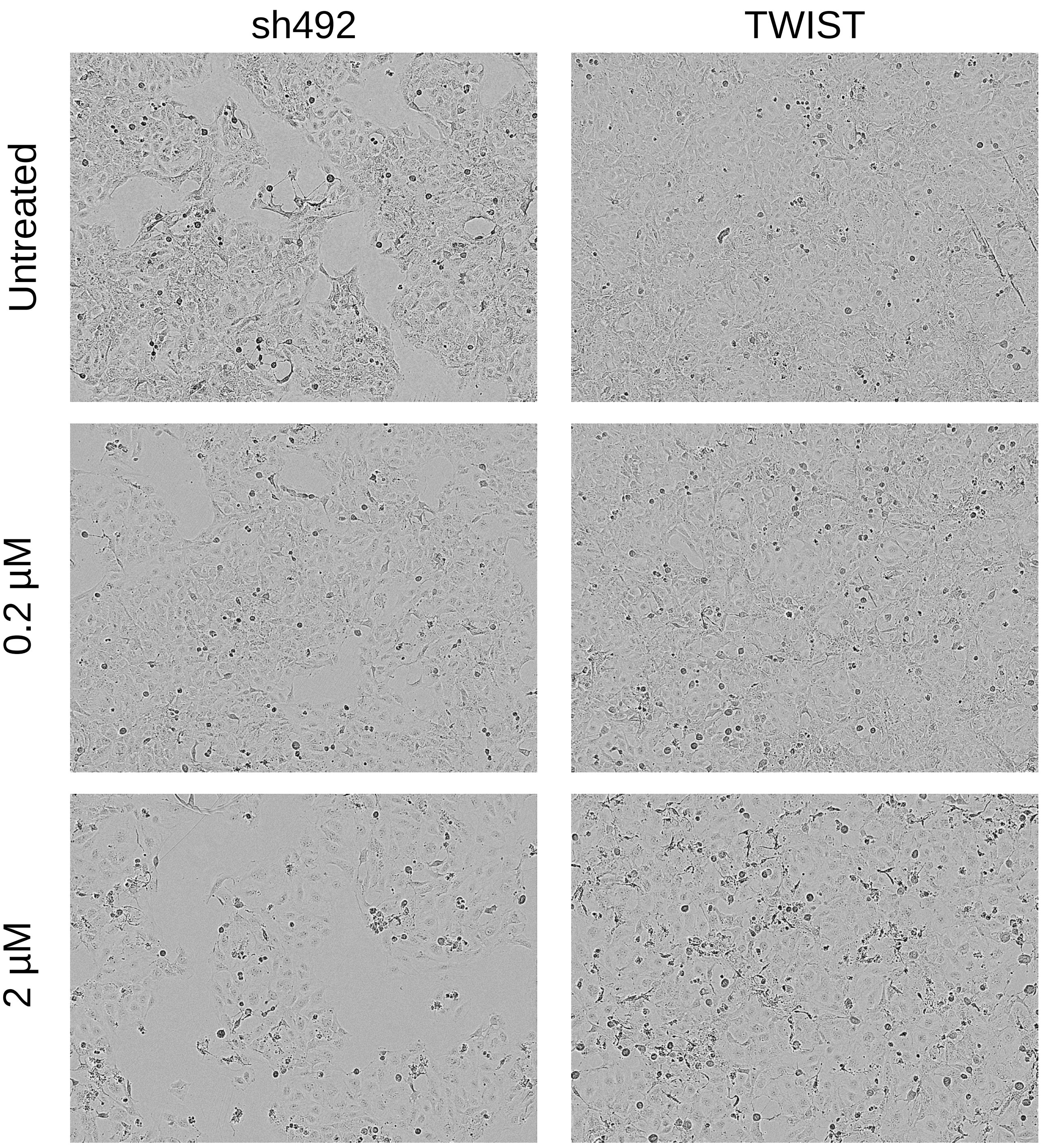

Figure S2. Representative images of Ov8GFP-TWIST and Ov8GFP-sh492 cells acquired three days post treatment with the indicated doses of cisplatin. Images such as these were analyzed to determine confluence, which is graphed in Figure 1c.

Figure S3

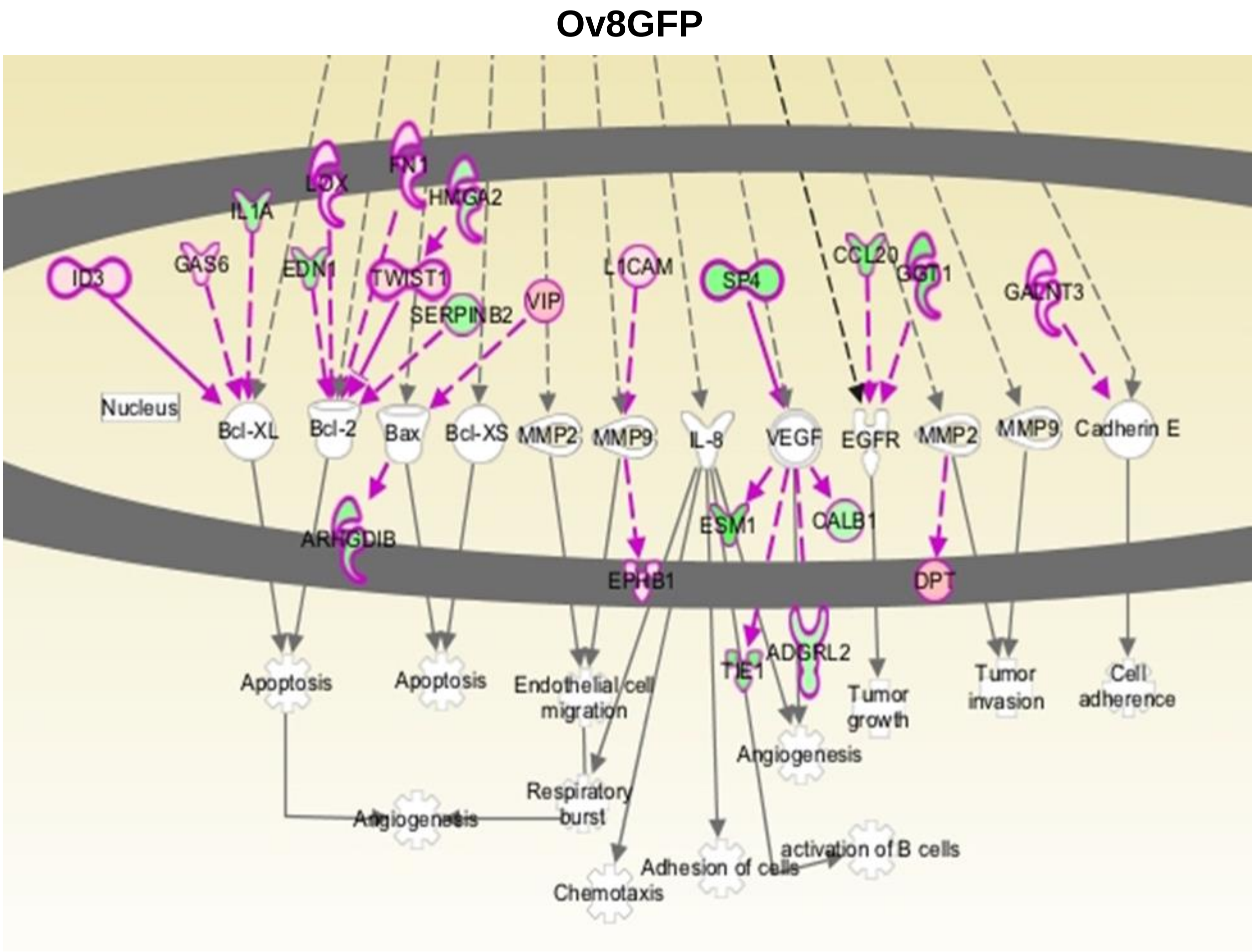

Figure S3. Ingenuity Pathway Analysis revealed that TWIST1 and known TWIST1 associated proteins such as IL-8, MMP2, MMP9, and E-cadherin lie in common pathways intersecting apoptosis and tumour growth and invasion. L1CAM and HMGA2, two TWIST1 associated genes identified by our RNA seq analysis, also appear.

Figure S4

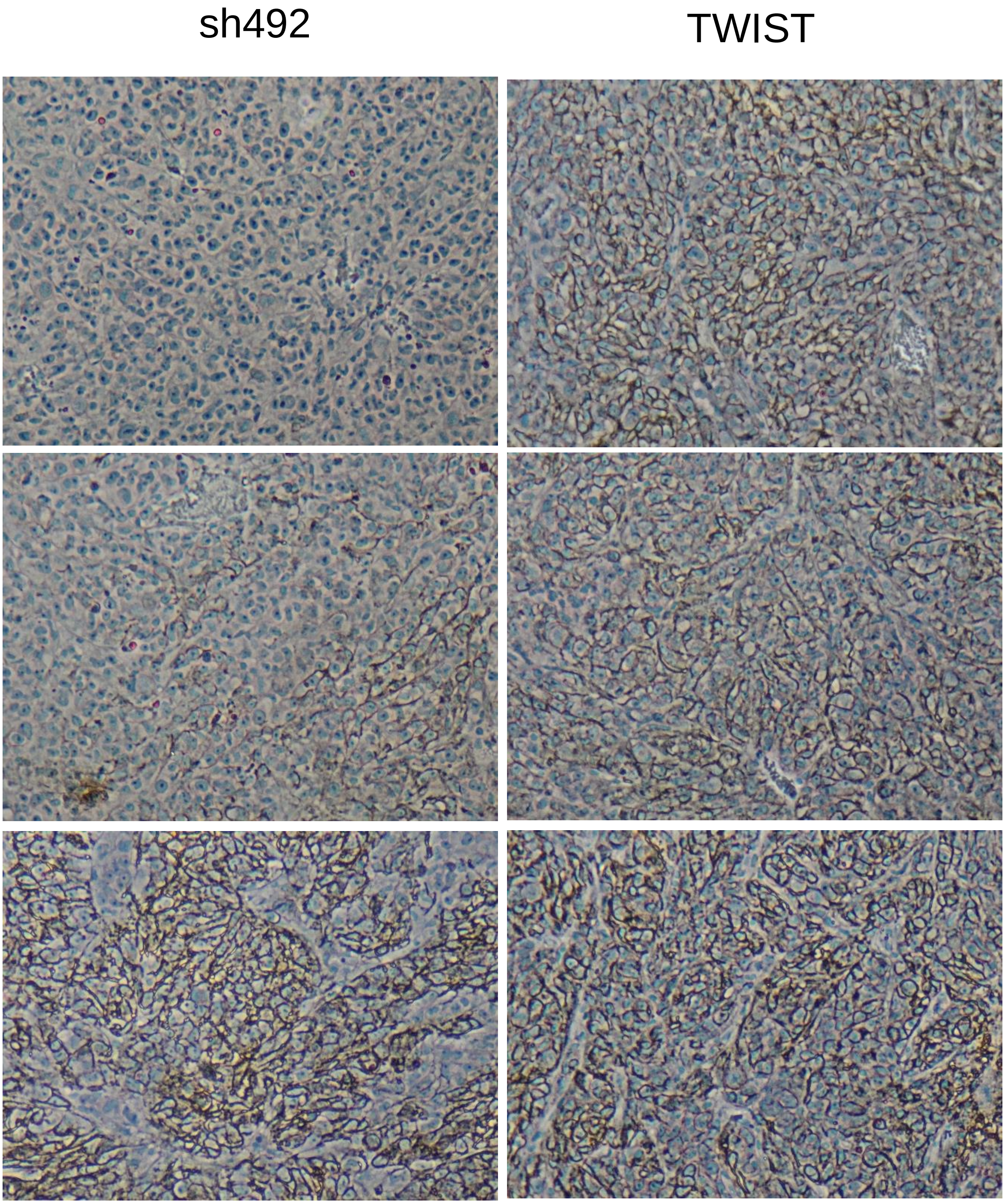

Figure S4. Representative images of Ov8GFP-sh492 and Ov8GFP-TWIST1 tumours from mice, immunostained for cell surface L1CAM. Ov8GFP-sh492 tumours were heterogeneous, with different tumour masses displaying no staining (top), light staining (middle) or dark staining. TWIST1 tumours were stained more uniformly and were typically dark with some lighter areas.

Figure S5

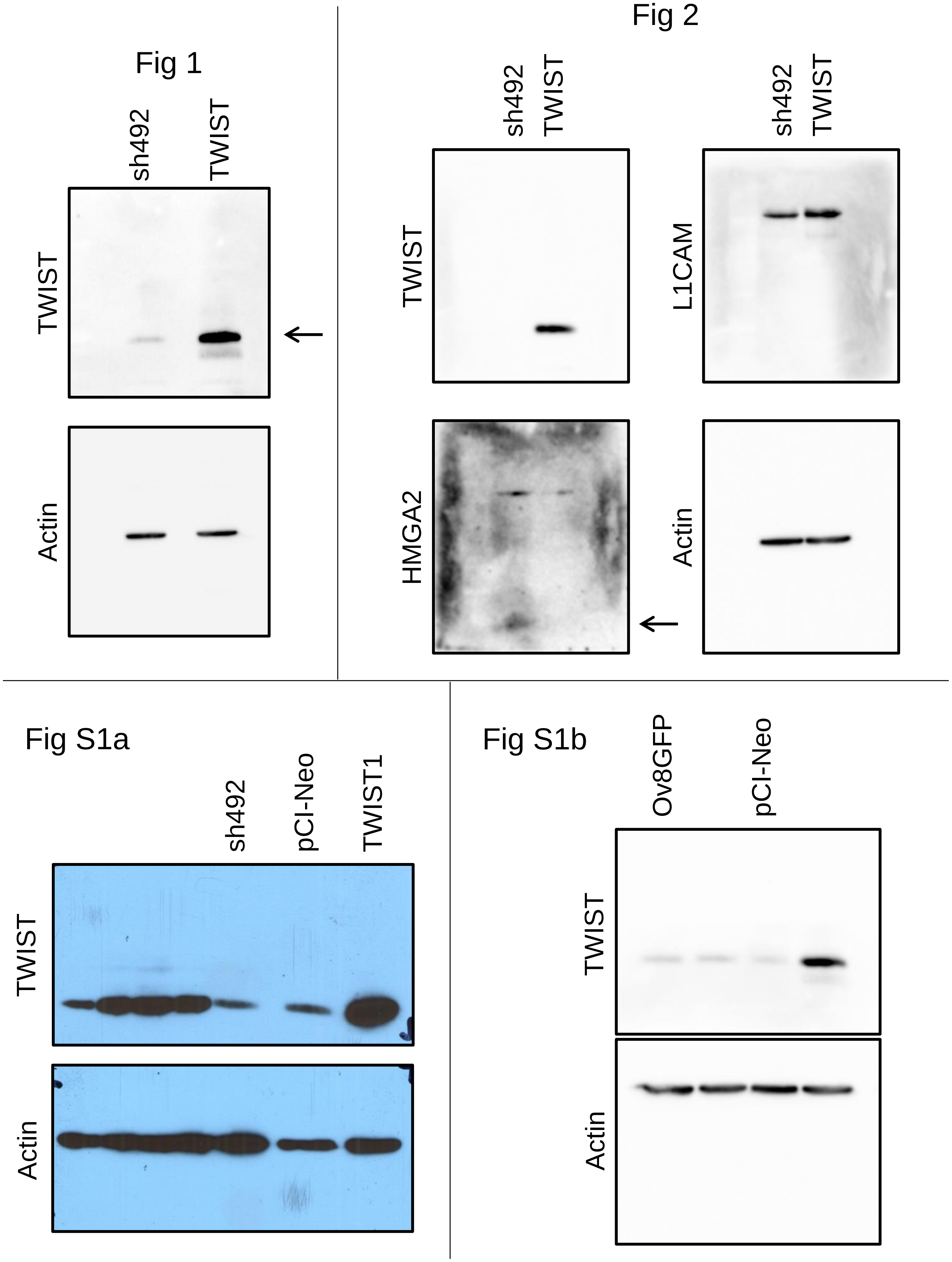

Figure S5. Full-length blots for the indicated figures from the main text, plus Figure S1.

Table S1

Gene	Expression		Log2 Fold Change	p Value	Function
	sh492	TWIST			
HCLS1	1.9192	0.3462	-2.4710	5.00E-05	Antigen signaling
ESM1	19.8290	5.3762	-1.8829	5.00E-05	Proangiogenesic
GGT1	7.5759	2.6660	-1.5068	5.00E-05	Glutathione metabolism
TIE1	4.2598	1.5563	-1.4526	5.00E-05	Endothelial adhesion molecule
CCL20	21.4373	7.9192	-1.4367	5.00E-05	Downstream of IL6/NFkB/STAT3 - immune attractant
ABCA1	1.7954	0.7054	-1.3478	5.00E-05	Cholesterol homeostasis
SP4	10.2972	4.1698	-1.3042	5.00E-05	Primarily neural transcription factor
ARHGDIB	8.3662	3.4472	-1.2792	1.50E-04	Microenvironmental communication to inhibit metastasis
LSAMP	0.6617	0.2736	-1.2739	2.50E-04	Neuron growth
MIR484,NDE1	36.8636	15.6657	-1.2346	1.50E-04	MicroRNA
EDN1	11.9078	5.0866	-1.2271	5.00E-05	Endothelin
IL1A	6.1556	2.7691	-1.1525	2.00E-04	Proinflammatory cytokine
CTPS2	10.0673	4.8606	-1.0505	1.50E-04	CTP synthase
SERPINB2	57.3030	28.1986	-1.0230	5.00E-05	Protease inhibitor, antiapoptotic
HMGA2	17.6553	8.9826	-0.9749	1.00E-04	DNA binding, chromosome condensation, DNA repair regulator
LOC643201	18.1145	9.4289	-0.9420	1.50E-04	Pseudogene
LPHN2	34.4611	18.9748	-0.8609	1.50E-04	Adhesion linked GPCR
CALB1	68.4107	38.3483	-0.8351	1.00E-04	Cytosol calcium buffering
FAM129A	9.9252	17.6015	0.8265	2.50E-04	ER stress response, regulates translation
DPYSL3	11.3916	20.8860	0.8746	5.00E-05	L1CAM pathway member, putative migration function
GAS6	21.2476	39.7292	0.9029	1.00E-04	Growth/adhesion/migration/survival. Interacts with Axl
CHN1	11.6973	22.1486	0.9210	5.00E-05	Lipid stimulated GTPase
GREM1	24.7823	47.6102	0.9420	5.00E-05	BMP inhib, proFGF signaling
SHISA9	7.0954	13.6759	0.9467	2.00E-04	Synaptic protein
EPHB1	8.8684	17.2622	0.9609	1.00E-04	Adhesion/migration signaling. ERK/JNK, possibly angiogenesis
RHOBTB1	1.7298	3.4910	1.0130	5.00E-05	GTPase involved with actin skeleton
L1CAM	17.3307	37.0653	1.0967	5.00E-05	Cell adhesion, migration in drug resistant cancers
PCSK9	4.3111	9.2437	1.1004	5.00E-05	Cholesterol homeostasis
OAS3	1.3659	2.9771	1.1240	1.50E-04	RNA synthesis inhibitor
LOX	4.5291	10.5536	1.2204	5.00E-05	Crosslinks ECM
DOK7	8.9338	21.3466	1.2567	5.00E-05	Neuromuscular interface
ID3	17.8285	43.0758	1.2727	5.00E-05	Inhibitor of bHLH binding
LEPREL1	8.2163	21.0238	1.3555	5.00E-05	Collagen assembly and linkage
CAPN6	2.0987	5.5978	1.4153	5.00E-05	May inhibit apoptosis, promote angiogenesis
FN1	225.0440	620.2980	1.4628	1.50E-04	Fibronectin
COL12A1	22.0742	61.1653	1.4704	5.00E-05	Collagen 12 alpha 1
AMIGO2	1.3070	3.9771	1.6055	5.00E-05	Cell-cell communication, neg regulator of apoptosis
GALNT3	0.3538	1.1292	1.6743	1.50E-04	Oligosaccharide biosynthesis
COL4A4	1.2217	4.2168	1.7873	5.00E-05	Collagen alpha 4
HOXA3	0.3283	1.1875	1.8548	5.00E-05	Developmental transcription factor - angiogensis and patterning
ATOH8	0.2612	0.9560	1.8718	5.00E-05	bHLH developmental TF
GDF6	0.4460	1.8793	2.0753	5.00E-05	Bone development - BMP signal responsive
PXDNL	0.5750	2.4422	2.0866	5.00E-05	Oxidative stress responsive endonuclease
MIR1909,REXO1	12.5434	53.5919	2.0951	5.00E-05	MicroRNA
MIR5193,UBA7	0.7722	5.1067	2.7254	5.00E-05	MicroRNA
BDKRB1	0.4423	3.1086	2.8133	5.00E-05	Receptor for bradykinin, an inflammatory vasodilator
LINC00452	0.2309	2.0640	3.1600	5.00E-05	Noncoding RNA
TWIST1	20.2035	209.0290	3.3710	5.00E-05	EMT, angiogenesis, metastasis, stem cell phenotype
MIR4324,SLC6A16	0.7451	21.5666	4.8552	5.00E-05	MicroRNA
VIP	0.0000	0.4792	N/A	5.00E-05	Vasodilator, also involved in survival
DPT	0.0000	1.2281	N/A	5.00E-05	ECM protein involved with TGFb
KCNA10	0.0000	0.2646	N/A	5.00E-05	Voltage gated potassium channel

Table S1. Complete list of differentially expressed genes output by RNA-seq analysis. Genes are ordered according to $\log_2$ fold change; genes downregulated when TWIST1 is overexpressed appear in green, and those upregulated appear in red. TWIST1 itself and genes selected for further study are shown in darker shades.
